# College and Hospital Notes

**Published:** 1907-07

**Authors:** 


### COLLEGE AND HOSPITAL NOTES.

The Buffalo General Hospital Training School for Nurses held its annual commencement exercises Tuesday evening, June 11, 1907.

The graduating class was composed of the following-named: Annie E. Munro, Florence P. Phillips, Catherine MacDonald, Laura Mildred Margaret Conlin, Jennie Scott Brewer, Ethelwyn E. Harris, Catherine Louise Campbell, Annie Gillespie, Annie Knox, Christine W. Fritz, Margaret E. Chapman, Nellie Jean Tench, Claire May Free, Beula Marmion Shaw, M. Sydna Sheldon, Catherine L. Burns, Emma Butler, Elizabeth Crysler and Estelle L. Cott.

The Reverend Robert Guthrie Freeman, pastor of the Lafayette Avenue Presbyterian Church, made the address, congratulating the school on its 28th commencement. Charles W. Pardee, president of the board of trustees, presented the diplomas to the class and Dr. William C. Krauss gave the school pins to the young women.

---

The annual commencement exercises of the Nurses' Training School of the W. C. A. Hospital, Jamestown, N. Y., were held Monday evening, May 27, 1907. The principal addresses were given by the Reverend Dr. Hickman and Dr. H. A. Eastman.

The graduates were Harriette M. Bray, Mrs. Cora B. Barnard, Mary C. Anderson, Mrs. Eliza McLellan, Alba E. Nethercott and Seruda Mitchell.

